# Nurses and the Unemployment Act

**Published:** 1920-11-13

**Authors:** 


### Nurses and the Unemployment Act.

The Minister of Labour was asked by Major Hamilton the position of nurses in general, and particularly district nurses, while under agreement to Queen Victoria's Jubilee Institute or a county nursing association, or candidates in training, under the provisions of the Unemployment Act. Sir M. Barlow replied that nurses employed under contract of service, or under contract of apprenticeship with money payment, come under the provisions of the Act, subject to the exceptions under Part II. of the First Schedule. The question whether these exceptions apply to any particular case or class of case is, under the Act, to be determined by the Minister of Labour, and is at present under consideration. The particular classes of nurses referred to are insurable, and candidates in training with pocket money, if it is regarded as payment for services rendered, are considered to be apprentices with money payment, and are insurable.

